# The marsupial trypanosome *Trypanosoma copemani* is not an obligate intracellular parasite, although it adversely affects cell health

**DOI:** 10.1186/s13071-018-3092-1

**Published:** 2018-09-20

**Authors:** Crystal Cooper, R. C. Andrew Thompson, Paul Rigby, Alysia Buckley, Christopher Peacock, Peta L. Clode

**Affiliations:** 1 Centre for Microscopy, Characterisation and Analysis, The University of Western Australia, Crawley, Western Australia 6009 Australia; 2 Central Analytical Research Facility, Queensland University of Technology, Brisbane, Queensland 4000 Australia; 3 School of Veterinary and Life Sciences, Murdoch University, Murdoch, Western Australia 6150 Australia; 4 Marshall Centre, School of Pathology and Laboratory and Medical Sciences, University of Western Australia, Crawley, Western Australia 6009 Australia; 5 UWA School of Biological Sciences, The University of Western Australia, Crawley, Western Australia 6009 Australia

**Keywords:** Host-parasite interactions, *Trypanosoma cruzi*, Marsupials, Australia, *Trypanosoma copemani*

## Abstract

**Background:**

*Trypanosoma cruzi* invades and replicates inside mammalian cells, which can lead to chronic Chagas disease in humans. *Trypanosoma copemani* infects Australian marsupials and recent investigations indicate it may be able to invade mammalian cells *in vitro*, similar to *T. cruzi*. Here, *T. cruzi* 10R26 strain (TcIIa) and two strains of *T. copemani* [genotype 1 (G1) and genotype 2 (G2)] were incubated with marsupial cells *in vitro*. Live-cell time-lapse and fluorescent microscopy, combined with high-resolution microscopy (transmission and scanning electron microscopy) were used to investigate surface interactions between parasites and mammalian cells.

**Results:**

The number of parasites invading cells was significantly higher in *T. cruzi* compared to either genotype of *T. copemani*, between which there was no significant difference. While capable of cellular invasion, *T. copemani* did not multiply in host cells *in vitro* as there was no increase in intracellular amastigotes over time and no release of new trypomastigotes from host cells, as observed in *T. cruzi*. Exposure of host cells to G2 trypomastigotes resulted in increased host cell membrane permeability within 24 h of infection, and host cell death/blebbing was also observed. G2 parasites also became embedded in the host cell membrane.

**Conclusions:**

*Trypanosoma copemani* is unlikely to have an obligate intracellular life-cycle like *T. cruzi*. However, *T. copemani* adversely affects cell health *in vitro* and should be investigated *in vivo* in infected host tissues to better understand this host-parasite relationship. Future research should focus on increasing understanding of the *T. copemani* life history and the genetic, physiological and ecological differences between different genotypes.

**Electronic supplementary material:**

The online version of this article (10.1186/s13071-018-3092-1) contains supplementary material, which is available to authorized users.

## Background

Trypanosomes are flagellate protozoan blood parasites responsible for a number of important, neglected diseases in the developing world. *Trypanosoma cruzi*, the causative agent of Chagas disease affects 6–7 million people and kills around 15,000 each year, mainly in Central and South America [1, 2]. There are concerns Chagas disease will become a worldwide health problem due to international migration, urbanisation, deforestation, poor disease detection, limited response to trypanocidal drugs, an increase in competent vectors, and insecticide resistance in vectors [3–5]. In Australia, *T. cruzi* was estimated to infect at least 3000 Latin American immigrants in 2006 [3, 6]. In Australia, there are two known trypanosome species, *T. noyesi* [7] and *T. teixeirae* [8], that are phylogenetically positioned within the *T. cruzi* clade. It is therefore possible that the vectors of these two species, which are currently unknown, could also spread the closely-related *T. cruzi* [9]. Recently it was found that bedbugs can transmit *T. cruzi* mechanically [5], when previously only reduviid bugs had been recognised as vectors. This suggests other invertebrates could also become mechanical vectors [9]. In a single study in Australia, native possums and a short-beaked echidna (*Tachyglossus aculeatus*) experimentally infected with *T. cruzi* resulted in a 60% mortality rate [10], demonstrating Australian marsupials are highly susceptible to *T. cruzi* infection. In South America, marsupials are natural reservoirs of *T. cruzi* increasing the number of animals infected and consequently the vectors, which creates spill-over into human populations [11].

The ability to invade cells, which leads to chronic infection with *T. cruzi*, has only been observed in a few species of trypanosomes. No other *Trypanosoma* spp. other than *T. cruzi* have been observed completing an intracellular life-cycle *in vivo*, except *T. dionisii* which is infective to bats both *in vitro* and *in vivo* [12–14]. Other trypanosomes that exhibit intracellular behaviour *in vitro* include *T. erneyi* [15], *T. theileri* [16], possibly *T. rangeli* [17–19], and *T. copemani* [20, 21]. *Trypanosoma copemani* is the only trypanosome from Australia that has been observed inside mammalian cells, and it has been implicated in the decline of an endangered marsupial species [20, 21]. Woylies (brush-tailed bettongs, *Bettongia pencillata*) infected with two strains of *T. copemani* [genotype 1 (G1) and genotype 2 (G2)] commonly showed signs of inflammation in various organs and *T. copemani* DNA was isolated from a number of different woylie tissues [20, 22, 23]. The morphological form of *T. cruzi* present inside the cell is the amastigote, which has a short internalised flagellum and undergoes division inside mammalian cells [24]. Structures suggestive of amastigotes were observed histologically in woylie heart tissue; however, immunochemistry was not used to determine with any certainty what species these amastigote-like cells belonged to [20]. Furthermore, G2 was reported to have intracellular stages that resembled amastigotes *in vitro* in various immortalised mammalian cell lines [Vero (African green monkey kidney epithelial cells), L6 (*Rattus norvegicus* skeletal muscle cells), HCT8 (*Homo sapiens* colon cells) and THP1 (*Homo sapiens* leukemic monocyte)] with the highest infection rate observed in Vero cells [21]. Botero et al. [21] proposed a possible life history for *T. copemani* that resembles that of *T. cruzi* based on their observations. However, to date the mechanisms by which *T. copemani* enters a cell is not known, and multiplication within host cells has not been observed. Additionally, the morphological form of *T. copemani* that is inside the host cell remains unconfirmed [21].

Due to the occurrence of mixed infections with both G1 and G2 of *T. copemani* in the woylie, further investigation is required to confirm if only one genotype is invading cells and what mechanisms are being utilised. Cell invasion processes used by *T. cruzi* are complicated and not entirely understood [1]. Depending on the strain of *T. cruzi* and the host cell infected [25, 26] a number of endocytic pathways involving various molecules are used by *T. cruzi* to gain entry into cells, although cells are not damaged upon entry [27–29]. It is known that *T. cruzi* recruits host cell lysosomes in order to build itself a parasitophorous vacuole after gaining entry to the cell [30]. The lysosomes fuse to the phagosome after internalisation of the parasite [31–33]. Following entry, *T. cruzi* transforms into the amastigote form and goes through roughly nine divisions (depending on the strain of parasite and host-cell used) before the amastigotes transform into trypomastigotes and escape the cell [34].

The objective of the present study was to investigate host cell-parasite interactions of *T. cruzi* 10R26 strain (TcIIa), *T. copemani* G1 and *T. copemani* G2 with marsupial immortalised potoroo kidney epithelial cells (PtK2) to investigate the presence of an intracellular life-cycle in *T. copemani* similar to that proposed by Botero et al. [21]. Vero cells were also investigated due to the high infection rate observed with this cell line *in vitro* [21]. Confocal microscopy and electron microscopy techniques, transmission (TEM) and scanning (SEM) electron microscopy, were used to observe how the cell membrane is affected by parasite attachment and to explore what interactions could be occurring at the host cell-parasite interface.

## Methods

### Maintenance of trypanosomes in culture

*Trypanosoma copemani* G1 and G2 were previously isolated from woylie blood samples and stored in liquid nitrogen at Murdoch University [20]. *Trypanosoma copemani* was grown in epimastigote form in an incubator at 28 °C with 5% CO_2_. Cultures were maintained in biphasic medium containing brain-heart infusion (BHI), BBL agar grade A, 0.48% gentamicin, and 10% defibrinated rabbit blood as a solid phase, and RPMI 1640 (Roswell Park Memorial Institute 1640) supplemented with tryptose (TRPMI) as in Noyes et al. [35] as a liquid phase. All liquid media was supplemented with 10% FCS and 1% penicillin-streptomycin. *Trypanosoma cruzi* 10R26 strain (TcIIa) was maintained at 37 °C with L6 cells and RPMI 1640 supplemented with 10% fetal calf serum (FCS) and 1% penicillin-streptomycin. *Trypanosoma cruzi* 10R26 strain was originally isolated by the research group of Michel Tibayrenc at the Institut de Recherce pour le Développement in Montpellier, France and was kindly provided to us by Professor Michael A. Miles.

### PtK2 cell infection kinetics of *Trypanosoma cruzi* 10R26 strain (TcIIa) and *T. copemani* G1 and G2

Monolayers of Vero or PtK2 (ATCC® CCL-56™) [36] cells were grown in 12.5 cm^2^ flasks, trypsinised at confluency, and seeded onto tissue culture-slides (16-wells) at a concentration of 1 × 10^4^ cells/ml. Vero cells were grown in RPMI 1640 supplemented with 10% FCS and 1% penicillin-streptomycin, and PtK2 cells were grown in minimal essential medium (MEM) supplemented with 10% FCS and 1% penicillin-streptomycin. After 24 h, the media was discarded to remove non-adherent cells, the cells washed with 1× phosphate-buffered solution (PBS), and 100 μl of parasite suspension containing 1 × 10^5^ parasites/ml was added to each well (1:10 cell/parasite ratio). Trypomastigotes were generated by taking log phase cultures and growing them in Grace’s insect media [37] without FCS, which caused them to enter stationary phase. Stationary phase epimastigotes transform into trypomastigotes when resuspended in full media prior to incubation with cells at 37 °C. However, not all parasites transform into trypomastigotes. *Trypanosoma cruzi* 10R26 strain (TcIIa) was investigated in order to provide a control for experiments and to demonstrate that it can infect marsupial cells *in vitro*. *Trypanosoma cruzi* was maintained at 37 °C with L6 cells in order to harvest metacyclic trypomastigotes newly emerged from cells. Slides were incubated at 37 °C and 5% CO_2_, and after 24 h slides were washed in 1× PBS before being resuspended in new MEM media. At different time-points post-infection (1, 6, 12, 24, 48, 72, 96 and 120 h), the supernatant was discarded, and slides were washed three times with 1× PBS to remove non-adherent parasites. Coverslips were removed, culture-slides were air-dried, fixed in methanol, stained with the commercial stain ‘Diff-Quik’, and mounted using Depex (Sigma-Aldrich, St. Louis, Missouri) for examination of intracellular parasites using light microscopy. Cell infectivity was measured by counting 300 cells from 10 wells in each slide. The number of cells infected and number of amastigotes or amastigote-like cells present in each infected cell were counted. Experiments were repeated on three separate occasions. Only cells that looked similar to *T. cruzi* amastigotes inside cells were counted. Attached parasites that were still in possession of a flagellum were excluded. Analyses of these data were performed using analysis of variance (ANOVA), and Fisher’s exact test in R 3.3.3 [38].

### Live-cell confocal microscopy of *Trypanosoma copemani* and PtK2 with propidium iodide

PtK2 cells were grown in 12.5 cm^2^ flasks, trypsinised at confluency, and seeded onto MatTek® (MatTek corporation, Ashland, Massachusetts) glass bottom dishes at a concentration of 1 × 10^4^ cells/ml. After 24 h cells were washed with 1× PBS and parasites were added at a concentration of 1 × 10^5^ cells/ml. Parasites were grown at 28 °C, and trypomastigotes for G1 and G2 were generated (see ‘PtK2 cell infection kinetics of *Trypanosoma cruzi* and *T. copemani* G1 and G2’). Parasites that were incubated with cells included *T. copemani* G2 trypomastigotes, G2 epimastigotes and G1 trypomastigotes. Controls included *T. copemani* G2 dead cells (heat treated at 72 °C overnight and re-suspended in fresh MEM), transformation media without parasites (Grace’s without FCS) and cell media without parasites (MEM). Propidium iodide (PI) was added to full culture medium at 200 μg/ml, 8 h after infection. Time-lapse images were taken at 15–60 min intervals in 3 x-y positions and 3 z positions in the culture dish, for up to 24 h. PI becomes fluorescent when it binds to DNA but it is not cell permeable and is therefore only fluorescent and clearly visible in cells with compromised membranes. All cells were counted in the field of view in order to estimate the proportion of cells with compromised membranes at 8, 16 and 24 h. Experiments were repeated twice on separate occasions. The difference between dead parasites and dead culture cells was easily observed at 20× magnification due to differences in the size and shape of cell nuclei (Additional file 1: Figure S1). Live-cell experiments were conducted using a Tokai Hit stage top incubation chamber on a Nikon A1Si Confocal (Tokyo, Japan), and image analysis was conducted using NIS AR elements and Image J. *Trypanosoma cruzi* could not be included in experiments due to lack of a quarantine approved laboratory (QC2) with a confocal microscope. Data analysis was performed using analysis of variance (ANOVA), and generalised linear mixed-effects models (*lme4*) in R 3.3.3 [38].

### Fixed cell confocal microscopy of *Trypanosoma cruzi* and *T. copemani* with PtK2 to observe lysosome recruitment

PtK2 cells were grown in 12.5 cm^2^ flasks, trypsinised at confluency, and seeded onto MatTek® glass bottom dishes at a concentration of 1 × 10^4^ cells/ml. After 24 h cells were washed with 1× PBS and parasites were added at a concentration of 1 × 10^5^ cells/ml. Parasites were grown at 28 °C, and trypomastigotes for G2 were generated (see ‘PtK2 cell infection kinetics of *Trypanosoma cruzi* and *T. copemani* G1 and G2’). For experiments investigating the recruitment of lysosomes to the site of attachment, Lysotracker® Deep Red (Molecular Probes® by Life Technologies, Waltham, Massachusetts) was added to full culture media containing live parasites and cells at a concentration of 75 nM for 5–10 min. Parasites (*T. cruzi*, G1 and G2) and cells were subsequently fixed after infection experiments using 4% paraformaldehyde in 1× PBS for 30 min followed by 3 washes in 1× PBS. After fixation, cells were stained with 300 nM DAPI for 5 min to visualise genetic material, then washed three times using 1× PBS. Following fixation cells were mounted with a coverslip using a low fade mounting media containing Tris-PO_4_ buffer (pH 7.6), polyvinyl alcohol, glycerol and chlorobutanol. Z-stacks were created using a Nikon A1Si Confocal. Nikon NIS AR elements and Image J were used to analyse the data. Experiments were repeated on three separate occasions. PtK2 cells and *T. cruzi* that were co-incubated for 72 h and stained with DAPI were used as a control to observe amastigotes inside PtK2 cells.

### Scanning (SEM) and transmission (TEM) electron microscopy

Cells (PtK2 and Vero) were grown on 10 mm round glass coverslips at 37 °C and infected with parasites for 24 or 48 h (as in ‘PtK2 cell infection kinetics of *Trypanosoma cruzi* and *T. copemani* G1 and G2’) before being processed for scanning electron microscopy (SEM). Cells without parasite infection were also prepared. Samples were prepared for SEM by fixation in 2.5% glutaraldehyde in 1× PBS and stored at 4 °C. Samples were dehydrated through a series of ethanol solutions (30, 50, 70, 90, 100, 100%) using a PELCO Biowave) then dried in liquid CO_2_ using a critical point drier. Coverslips were mounted on stubs with adhesive carbon and coated in 2 nm platinum (Pt) and 10 nm carbon. Trypanosomes were imaged at 3 kV using the in-lens secondary electron detector on a Zeiss 55VP field emission SEM. For transmission electron microscopy (TEM) cells were grown in 12.5 cm^2^ flasks, infected for 48 h, trypsinised, and pelleted before fixation. Samples were fixed in 2.5% glutaraldehyde in 1× PBS and processed using a PELCO Biowave, where samples were post-fixed in 1% OsO_4_ in 1× PBS followed by progressive dehydration in ethanol (30, 50, 70, 90, 100%) then acetone, before being infiltrated and embedded overnight at 70 °C in Procure-Araldite epoxy resin. Resin blocks were trimmed using razors followed by glass knives before sections 120 nm thick were cut using a diamond knife on a Leica microtome and mounted on copper grids. Digital images were collected from unstained sections at 120 kV on a JEOL 2100 TEM fitted with a Gatan ORIUS1000 camera.

## Results

### *Trypanosoma cruzi* 10R26 strain (TcIIa) infects PtK2 cells revealing a four day cell cycle

**Fig. 1:**
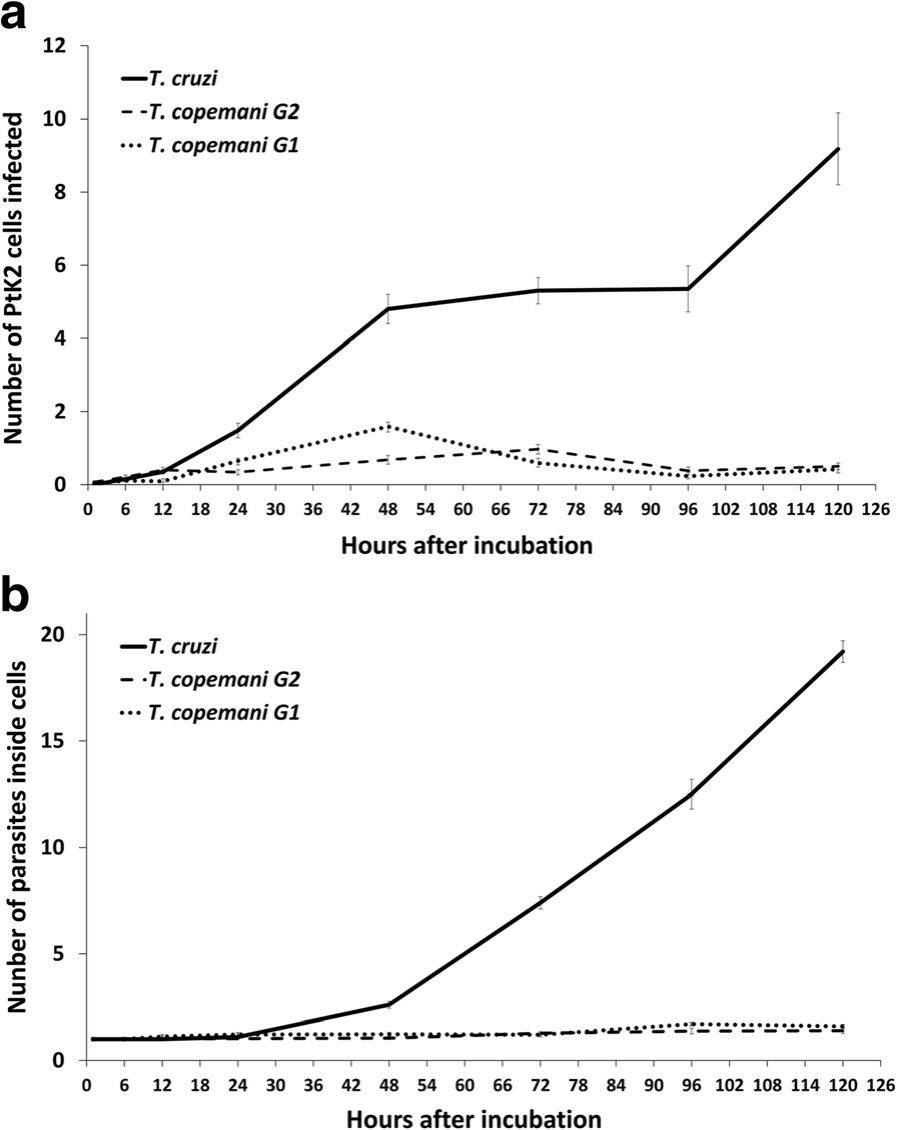
*Trypanosoma cruzi* and *T. copemani* G1 and G2 infecting potoroo kidney epithelial (PtK2) cells over 5 days. **a** Number of cells from 9000 (300 cells from 10 culture wells in 3 repeated experiments) (mean ± SE) infected with parasites over the five days. **b** Number of intracellular parasites inside 9000 cells (300 cells from 10 culture wells in 3 repeated experiments) (mean ± SE) in infected cells over five days

**Fig. 2:**
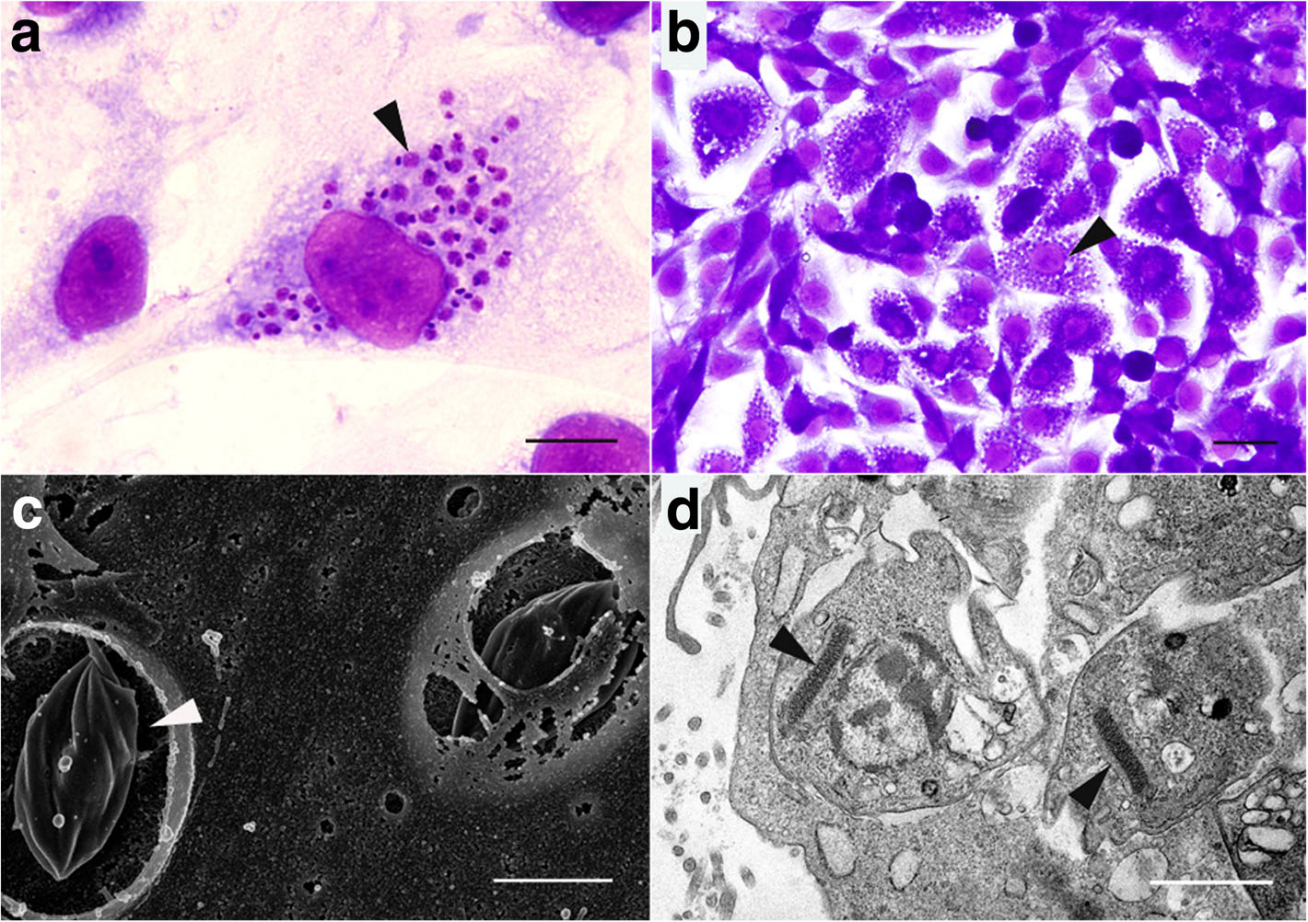
Microscopy of *Trypanosoma cruzi* infecting potoroo kidney epithelial (PtK2) cells. **a** Light micrograph of Diff-Quik stained cells and parasites after 96 h incubation showing a single cell with amastigotes inside, as recognisable by their round nucleus and disc shaped kinetoplast (arrowhead). **b** Heavily infected area of PtK2 cells (arrowhead) following incubation with *T. cruzi* for 120 h and stained with Diff-Quik. **c** Scanning electron micrograph of *T. cruzi* infecting PtK2 cells. The dying cell membranes were broken fortuitously exposing developing *T. cruzi* amastigotes (arrowhead) inside. **d** Transmission electron micrograph of *T. cruzi* amastigotes developing inside PtK2 cells. *T. cruzi* is recognisable by the dense elongate kinetoplasts (arrowheads). *Scale-bars*: **a**, 10 μm; **b**, 20 μm; **c**, 2 μm; **d**, 1 μm

The number of PtK2 cells infected with *T. cruzi* increased over time, although it remained below 10% over five days (Fig. 1). *Trypanosoma cruzi* showed an increase in the number of amastigotes inside cells over time due to the division of parasite cells. Infection of PtK2 cells with *T. cruzi* revealed approximately a four day intracellular cell cycle, evident from the increase in infected cells after 96 h incubation due to the release of new trypomastigotes from initially infected cells (Fig. 1). After 120 h it was too difficult to count infected cells due to the large number of parasites inside some cells, and an increase in healthy cells making the cell monolayer too thick to clearly observe (Fig. 2a, b). Scanning electron micrographs revealed developing *T. cruzi* amastigotes, which were able to be seen inside PtK2 cells after the breakdown of the cell membrane (Fig. 2c). Transmission electron micrographs showed healthy *T. cruzi* amastigotes inside cells, which were clearly recognisable by their dense kinetoplasts (Fig. 2d).

### *Trypanosoma copemani* G1 and G2 do not infect marsupial cells in a similar way to *T. cruzi*

**Fig. 3:**
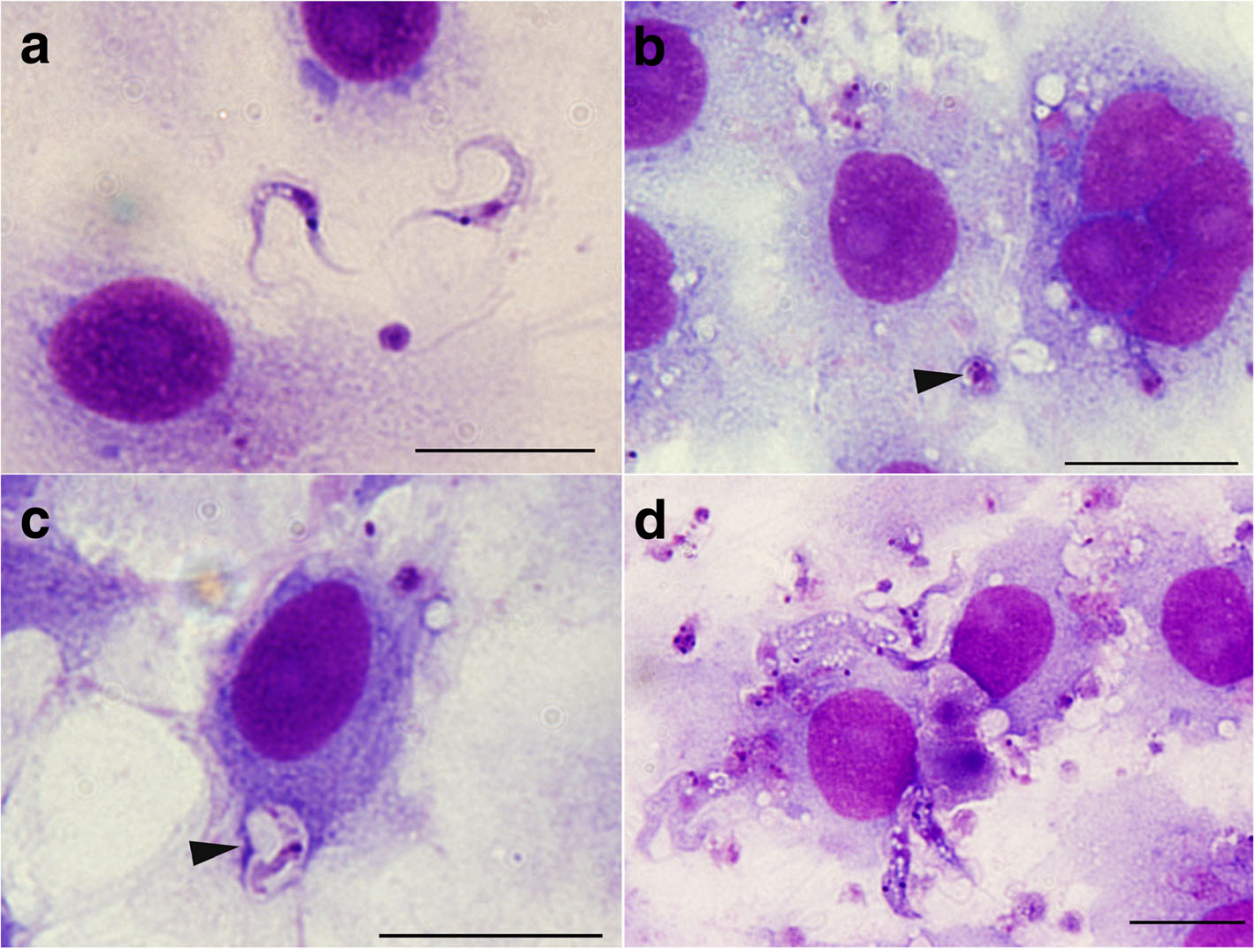
Microscopy of *Trypanosoma copemani* G2 incubated with potoroo kidney epithelial (PtK2) cells. **a** Light microscopy of G2 trypomastigotes. **b** Light microscopy of G2 amastigote-like cells inside PtK2 cells (arrowhead). **c** G2 trypomastigote inside a vacuole within the cell (arrowhead). **d** G2 attached to the outside of cells. All images are stained with Diff-Quik. *Scale-bars*: 20 μm

When compared to the results with *T. cruzi*, the number of cells infected with *T. copemani* was significantly lower and there was no increase in intracellular parasites over time (Fig. 1). The sum of infected cells with G1 was 334, G2 was 319, and *T. cruzi* was 2408 in total. ANOVA analysis indicated there was a statistically significant difference between group means of the number of cells infected with each parasite strain over time (G1, G2 and *T. cruzi*) (*F*_(14, 2136)_ = 58.8, *P* < 0.001). Tukey’s honest significance *post-hoc* test demonstrated *T. cruzi* was significant from G1 and G1 (*P* < 0.001), but G1 and G2 were not significant from each other (*P* = 0.96). G2 (Fig. 3a) infected cells most often as amastigote-like parasites when viewed using light microscopy (Fig. 3b) although, intracellular trypomastigotes were also observed, within a vacuole (Fig. 3c). Multiple parasites were routinely observed attached to the outside of cells (Fig. 3d), although PtK2 cells with large numbers of *T. copemani* on the cell membrane surface that had visible flagella and no halo or vacuole were excluded due to the likelihood that they were not intracellular (Fig. 3d). *Trypanosoma copemani* G1 also infected cells most often in what looked like the amastigote form when viewed using light microscopy (Additional file 2: Figure S2). The number of intracellular *T. cruzi* amastigotes in PtK2 cells greatly outweighed the number of intracellular G1 or G2 parasites (Fig. 1). ANOVA analysis demonstrated a statistically significant difference between group means of the number of amastigotes inside cells (*F*_(14, 3036)_ = 16.8, *P* < 0.001). Tukey’s honest significance *post-hoc* test demonstrated *T. cruzi* was significant from G1 and G1 (*P* < 0.001), but G1 and G2 were not significant from each other (*P* = 0.99).

### PtK2 cells increase uptake of propidium iodide over time in presence of *Trypanosoma copemani* G2 trypomastigotes

**Fig. 4:**
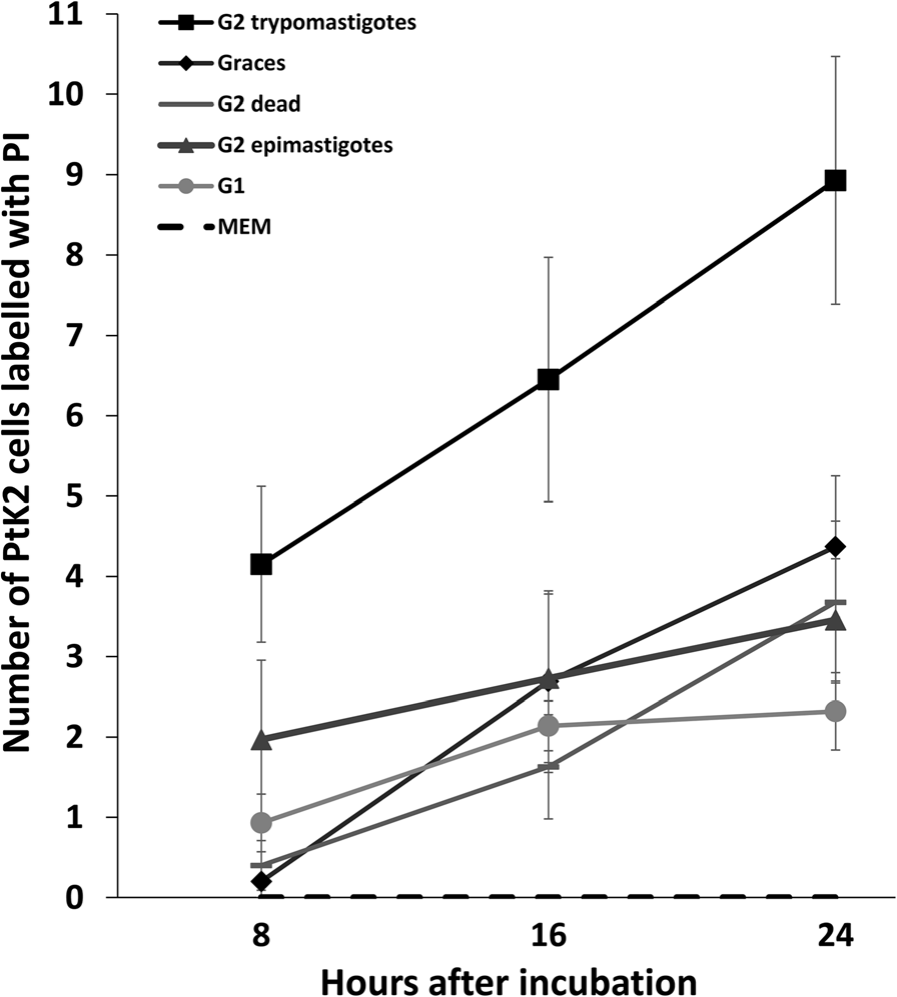
The number (mean ± SE) of potoroo kidney epithelial (PtK2) cells displaying propidium iodine (PI) fluorescence, and thus with permeabilised or compromised membranes, after incubation with *Trypanosoma copemani* for 24 h. *T. copemani* G2 trypomastigotes, G2 epimastigotes and G1 trypomastigotes were incubated with PtK2 cells in MEM media for 24 h. Controls included dead parasites (G2 trypomastigotes), PtK2 cells in MEM without parasites and PtK2 cells in Grace’s media without FCS or parasites. Cells in 3 separate fields of view were counted (20×) and experiments were repeated twice to gain the proportion of cells displaying PI fluorescence for each treatment

**Fig. 5:**
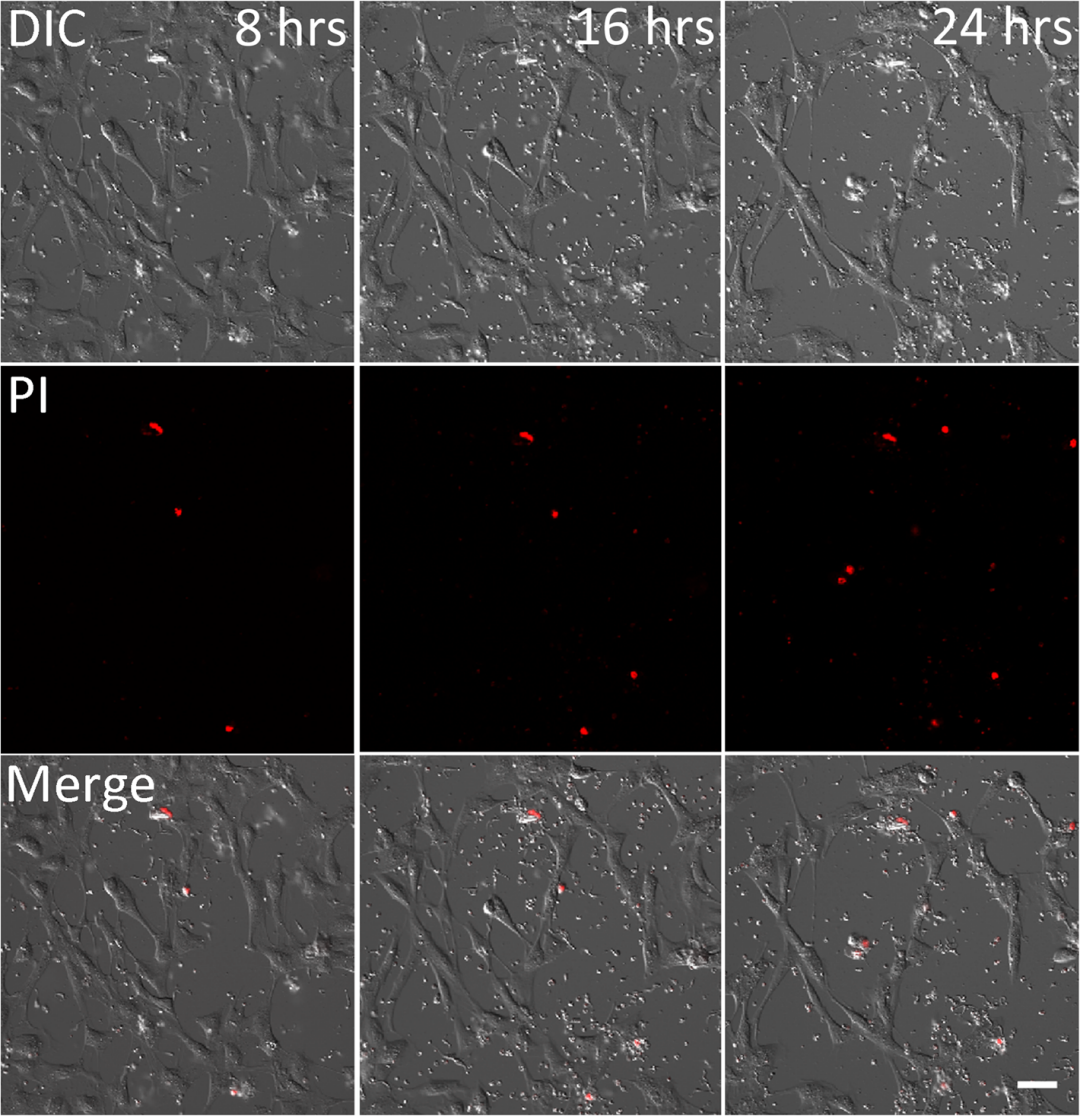
*Trypanosoma copemani* G2 and potoroo epithelial kidney (PtK2) cells incubated with propidium iodide (PI). Cell uptake of PI increases over time when exposed to G2 trypomastigotes: 8 h; 16 h; 24 h. Channels are split into differential interference contrast (DIC), PI, and both channels merged (Merge). See Additional file 1: Figure S1 and Additional file 4 for higher magnification figures and a live-cell time-lapse video. *Scale-bar*: 50 μm


There was an increase in PtK2 cells positive for PI staining when they were incubated with *T. copemani* parasites, but also when they were grown in Grace’s insect media without FCS or parasites (Fig. 4). In control experiments, healthy PtK2 cells grown in MEM with FCS and without parasites exhibited no increase in cells stained with PI (Fig. 4, Additional file 3). The addition of G2 trypomastigotes to the cell cultures resulted in an almost 3-fold increase in the percentage of cells that were positive for PI staining (Figs. 4, 5 and Additional file 4). ANOVA using linear mixed models (treating replicates and time points as random effects due to non-independence of PI uptake by cells over time) demonstrated that different treatments affect PI uptake in cells (*χ*^2^_(2)_ = 8.57, *P* = 0.013). Fisher’s exact test was used to demonstrate independence between the frequency of G2 trypomastigotes infecting cells and all other treatments (*P* < 0.001), with an odds ratio ranging between 2.19–3.50 indicating that G2 trypomastigotes were 2–3 times more likely to cause PI staining in cells. MEM media without parasites was also significantly different compared to all other treatments (*P* < 0.002–0.009) as no increase in PI staining was observed. The remaining interactions (G1 trypomastigotes, G2 dead, G2 epimastigotes and Grace’s without FCS or parasites) were not independent from one another under Fisher’s exact test. In some cases the cells were removed from the glass substrate when multiple parasites (G2 trypomastigotes) attached to the surface and occasionally the membrane of the cell burst (Additional file 4). Observations of cells incubated for a further 24 h following washing in 1× PBS and resuspension in MEM media demonstrated cell recovery in the next 24 h period (Additional file 5: Figure S3).

**Additional file 3:** Time-lapse live-cell video of a control experiment showing potoroo epithelial kidney (PtK2) cells in full MEM media supplemented with 10% FCS incubated with propidium iodide (PI) (red) and without parasites from 8 to 24 h after incubation. This control experiment demonstrates that healthy cells do not take up PI over time. Healthy cells can also be observed dividing. (AVI 51372 kb)



**Additional file 4:** Time-lapse live-cell video demonstration of potoroo epithelial kidney (PtK2) cells incubated with *Trypanosoma copemani* G2 from 8 to 24 h after incubation. Cell DNA stains with propidium iodide (red) over time when exposed to *T. copemani* G2 trypomastigotes due to increased cell membrane permeability. (AVI 54972 kb)


### Observation of the host cell-parasite interaction at the site of *Trypanosoma copemani* attachment

**Fig. 6:**
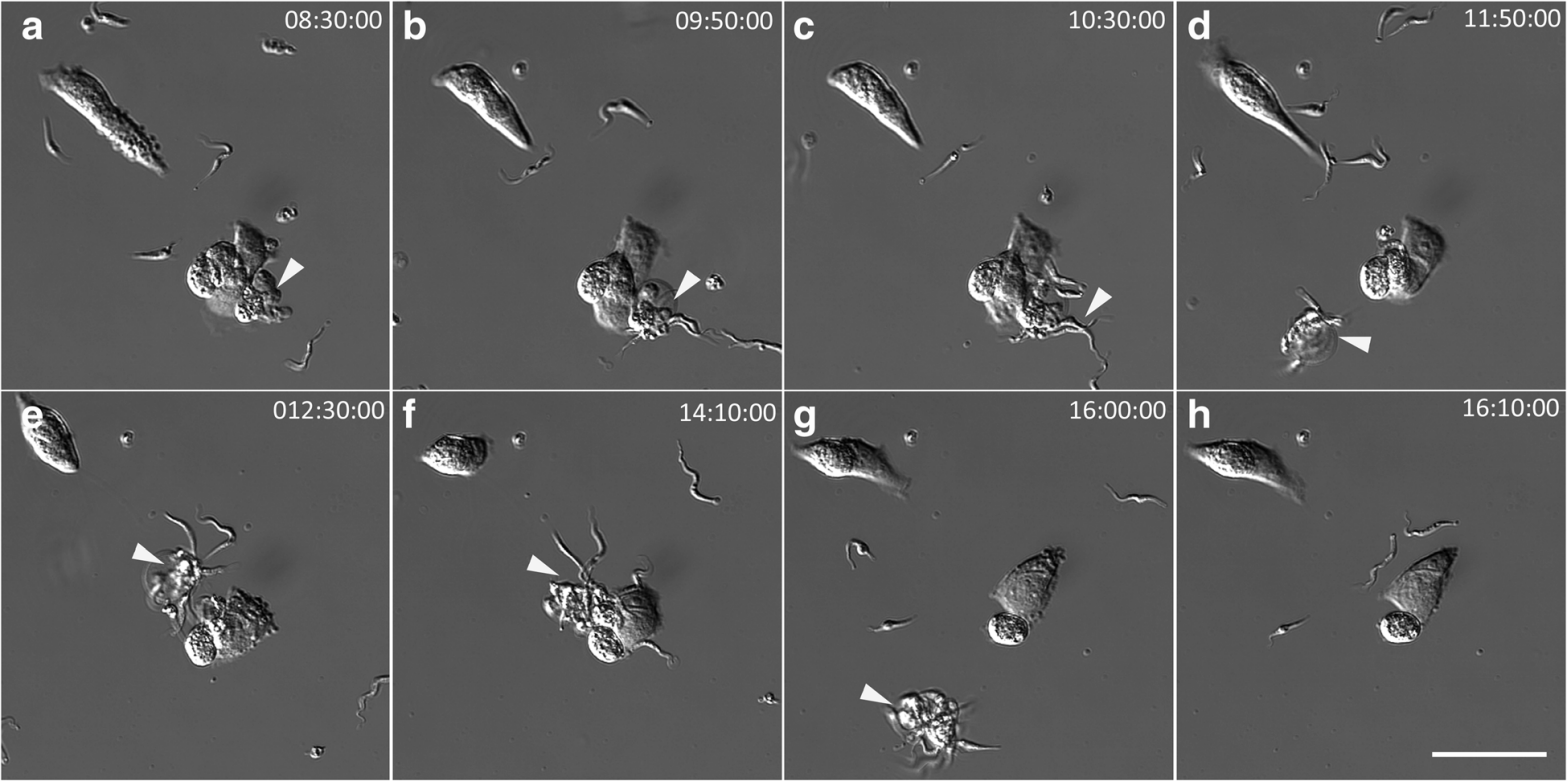
Multiple attachment of *Trypanosoma copemani* G2 trypomastigotes to Vero cells at different time-points during an 8 h incubation, 8 h after parasites were added to cells. The video shows parasite attachment can cause cell detachment from the glass substrate (indicated by arrowheads). Each panel **a**-**h** shows time after incubation with G2. See Additional file 6 for the live-cell time-lapse video. *Scale-bars*: 50 μm

**Fig. 7:**
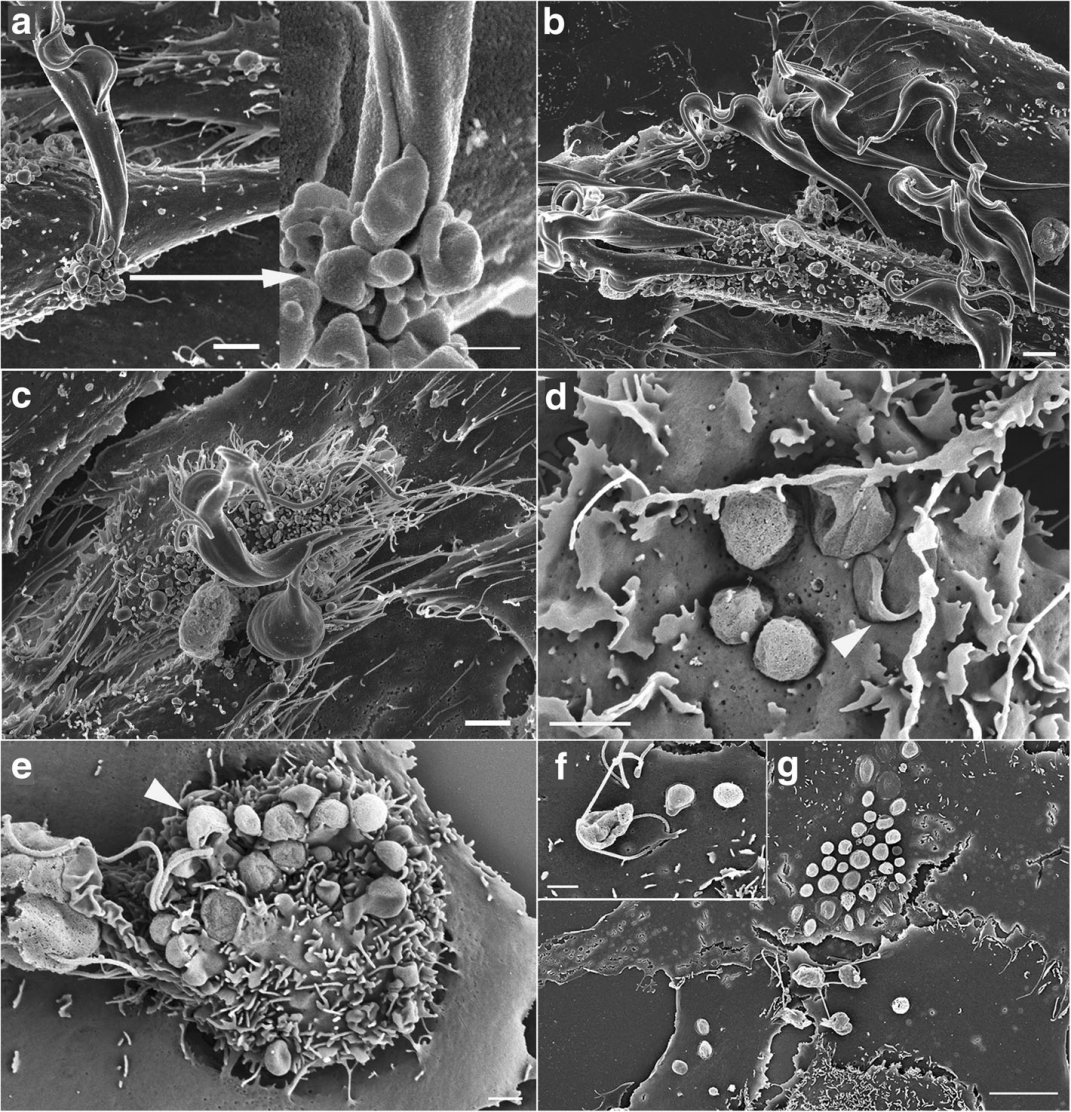
High resolution scanning electron micrographs of *Trypanosoma copemani* G2 trypomastigotes after incubation with Vero cells for 24 (**a**-**c**) and 48 h (**d**-**g**). **a** Parasite attached to cell that is blebbing around the cell attachment site and an arrow showing higher magnification of the same site. **b** Attachment of multiple parasites to a cell that appears to be blebbing. **c** Attachment of multiple parasites to a cell that appears to be blebbing. **d** Parasites embedded in the cell membrane. Arrow points to a visible flagellum, **e** Parasites embedded in the cell surface and some retain long flagella that extend out of the cell (arrow). **f** Parasite that appears to be degrading with a flagellum on the surface of a cell **g** Multiple parasites attached to the cell surface that appear to be degrading. *Scale-bars*: **a**, 2 μm and 1 μm; **b**-**f**, 2 μm; **g** 10 μm

**Fig. 8:**
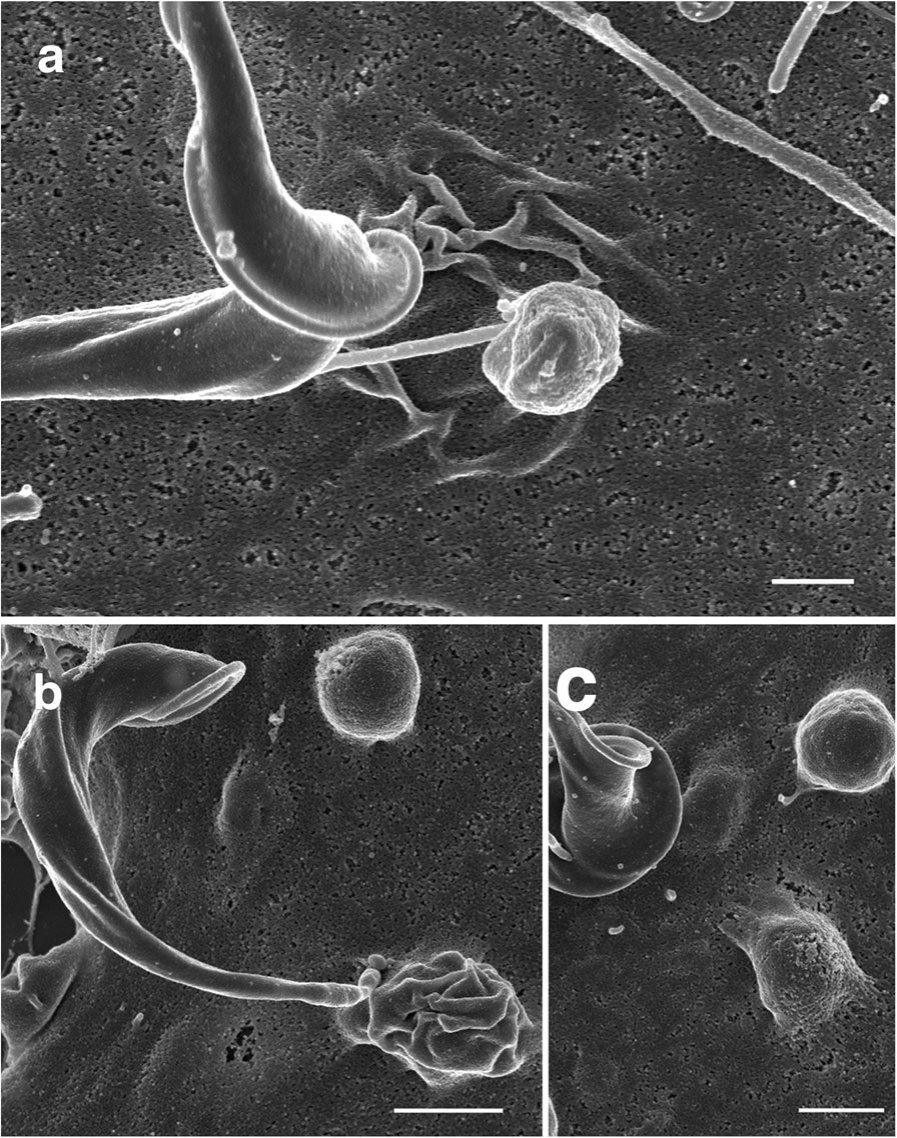
High resolution scanning electron micrographs of *Trypanosoma copemani* G2 after incubation with potoroo epithelial kidney (PtK2) cells for 24 h. **a** Attached *T. copemani* trypomastigotes and external amastigote attached to the cell surface. **b** Attached *T. copemani* trypomastigote. **c** Blebbing in PtK2 cell. *Scale-bars*: 1 μm

**Fig. 9:**
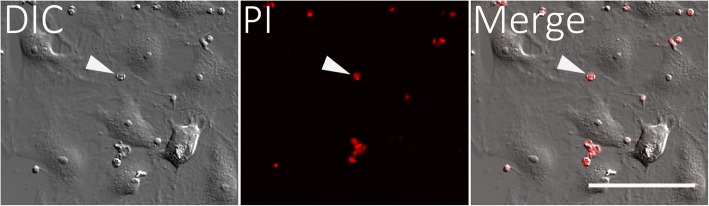
*Trypanosoma copemani* G1 after 24 h incubation with potoroo kidney epithelial cells and propidium iodide (PI). Amastigote-like cells are recognisable by the round appearance and the presence of PI indicates their membranes are compromised (arrow). Channels are split into DIC, PI, and both channels merged. *Scale-bar*: 50 μm


It was observed in time-lapse experiments that G2 trypomastigotes caused PtK2 cells to detach from the flask or to undergo blebbing (Fig. 6) and this was also observed in Vero cells (Fig. 6, Additional file 6). There was a noticeable host cell-parasite interaction at the cell membrane surface where G2 had come into contact with either Vero (Fig. 7) or PtK2 (Fig. 8) cells when imaged by SEM. Following 24 h incubation with parasites, Vero cells were observed blebbing where single (Fig. 7a), or multiple (Fig. 7b, c) parasites attached to a cell. Parasites were observed embedded in the cell membrane of Vero cells after 48 h where the cell membrane had partially engulfed the parasites (Fig. 7d). In some cells, multiple parasites became embedded occasionally with remaining flagella (Fig. 7e), although their membranes did appear damaged (Fig. 7F, G) compared to the healthy trypomastigotes (Fig. 7b). In PtK2 cells a number of interactions were observed including cell blebbing (Fig. 8a, b), circular-shaped masses (Fig. 8b, c) and trypomastigote attachment (Fig. 8a, b) on the surface of the cells after 48 h. After 24 h incubation with *T. copemani* trypomastigotes, cells were washed and re-incubated with PI. Amastigote-like parasites were observed labelled with PI and attached to the surface of cells. This was observed in G1 (Fig. 9) and G2 with PtK2 and Vero cells (data not shown) indicating the amastigote-like parasites membranes were damaged, consistent with the observations of degraded membranes seen in SEM images.

**Additional file 6:** Time-lapse live-cell demonstration that multiple attachment of *Trypanosoma copemani* G2 trypomastigotes to Vero cells can cause cell detachment from the glass substrate. Live-cell time-lapse video taken over 8 h. (AVI 81977 kb)


**Fig. 10:**
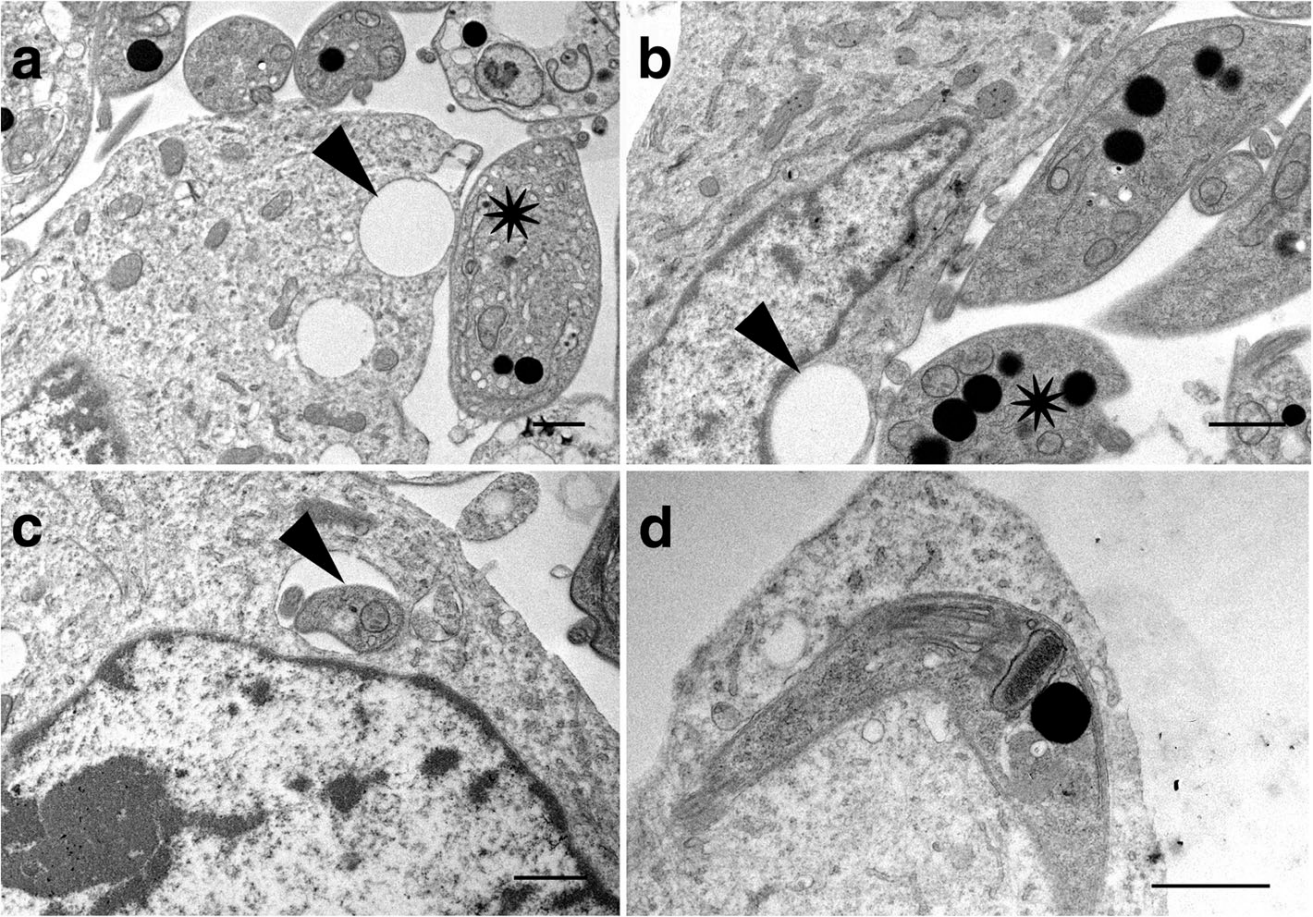
Transmission electron micrographs of potoroo kidney epithelial cells (PtK2) after incubation with *Trypanosoma copemani* G2 for 48 h. **a** Parasite attached to the surface of a cell. Parasite indicated by an asterisk is attached to the surface of the cell with a vacuole (arrow) underneath. **b** Two parasites attached to the surface of a cell one parasite (asterisk) with a vacuole underneath (arrow). **c** *T. copemani* inside a cell, inside a vacuole recognisable by the flagellum (arrow). **d** *T. copemani* attached to a cell, recognisable by the kinetoplast (arrow). *Scale-bars*: 1 μm

**Fig. 11:**
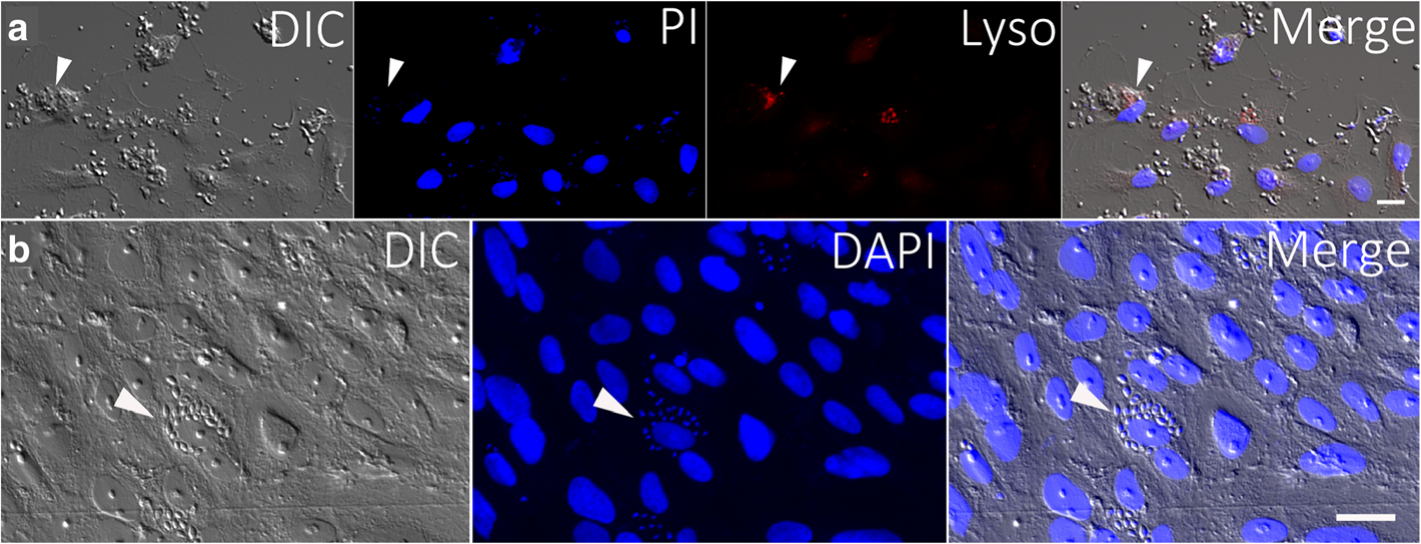
**a** *Trypanosoma copemani* infecting potoroo kidney epithelial (PtK2) cells *in vitro* after 24 h (fixed samples). Parasites and cells were stained with DAPI and Lysotracker®. DIC, DAPI, and Lysotracker® channels are split to show individual staining, and the merged panel is shown last. Arrows point to a cell with attached parasites and lysosomes that are not localised in the same place as parasite attachment. Cells are recognisable by their larger nuclei and parasites by their smaller nuclei stained with DAPI. **b** *Trypanosoma cruzi* infecting potoroo kidney epithelial (PtK2) cells *in vitro* after 72 h (fixed samples). Arrow indicates an infected cell. Parasites are recognisable by their smaller nucleus and kinetoplast stained with DAPI. Cells are distinguishable by their larger DAPI stained nuclei compared to the smaller parasite nuclei. Channels are split into DIC, DAPI and both channels merged. *Scale-bar*: 20 μm

TEM images revealed that when many parasites attached to the cultured cell surface a vacuole was often present within that cell, directly underneath the area of parasite attachment (Fig. 10a, b). Parasites were observed that appeared to be within the cell cytoplasm, occasionally within a clear vacuole (Fig. 10c) and the morphological form of G2 attached to the cell surface was often the trypomastigote (Figs. 7a, c and 10d). Lysosomes were not observed at the site of parasite attachment to cells or localised near parasites in PtK2 cells (Fig. 11a), or Vero cells (data not shown). *Trypanosoma cruzi* was used as a control to demonstrate the appearance of intracellular parasites (Fig. 11b). It is important to note that reservosomes or lysosome-like compartments inside the parasites can also take up Lysotracker®.

## Discussion

It is clear that the interactions observed between *T. copemani* and cultured mammalian cells differ from that seen in *T. cruzi*. Although there are some similarities, there is no evidence *T. copemani* is capable of completing its life-cycle inside cells. *Trypanosoma copemani* amastigote-like cells did not increase in number over time compared to *T. cruzi*, indicating no intracellular cell division of intracellular parasites. It is therefore unlikely that *T. copemani* has a similar life history to *T. cruzi*. *Trypanosoma copemani* does not enter marsupial cells (PtK2) at the same rate observed entering Vero cells [21]. Botero et al. [20, 21] found that Vero cells exhibited the highest infection rate with an estimated 70% of cells infected at 48 hours post-infection, while in three other cell lines it was between 7 and 15%, although cells were added at the same ratio but with a lower concentration (1.5 × 10^3^ cells/ml) than in the current study (1 × 10^4^ cells/ml).

There was an increase in PtK2 cell membrane wounding evident from cells expressing PI following exposure to either *T. copemani* parasites or when simply exposed to media without serum, as these treatments place the cells under stress. G2 trypomastigotes caused three times more cells to become positive for PI staining, which was likely due to their attachment to the cell membranes, which on occasion were further observed to be blebbing [39], becoming detached from the flask, or even bursting. It was interesting that the parasites which were resuspended in MEM (with 10% FCS) before being incubated with cells caused the same level of cell membrane damage to PtK2 cells as Grace’s media without 10% FCS, and that cell membrane wounding increased in the presence of either G1 or G2. The parasites (G1 and G2) appear to adhere to the cells in the same manner as *T. cruzi*, which also initially attaches to cells by their posterior tip or anterior flagellum [28]. However, *T. cruzi* enters cells by slipping into the membrane without damage and without causing the cells to burst. *Trypanosoma cruzi* results in positive staining of PI in cells when essential cell processes involved in cell wound repair are blocked [40]. In the process of cell entry by *T. cruzi* the invading parasites are surrounded by a membrane of host origin after entry and enclosed in a parasitophorous vacuole [27]. *Trypanosoma cruzi* takes advantage of the cellular repair mechanisms used by cells involving free Ca^2+^ and lysosome recruitment. However, it is only when Ca^2+^ is removed from the environment, cell membrane repair is interrupted and *T. cruzi* causes damage to the membrane resulting in positive PI staining [27]. *Trypanosoma cruzi* epimastigotes do not cause cell wounding, which might be due to the pore-forming protein, Tc-Tox, secreted by *T. cruzi* metacyclic trypomastigotes but not epimastigotes [41]. *Trypanosoma copemani* epimastigotes do not damage cellular membranes in the same manner as trypomastigotes, and the mechanism is not understood. G2 had a more pronounced interaction with cells and is genetically more distant from all other genotypes of *T. copemani* (including G1) that are found in other marsupials at two phylogenetically relevant loci (Gapdh and *18S* rDNA) [20, 42] and the mitochondrial kinetoplast DNA [43]. The morphology and behaviour of G1 and G2 appeared different in this study indicating that these genotypes may be sufficiently diverged to be considered different species, although further genetic analysis is required.

Although *T. copemani* often appears to be intracellular in the host cell cytoplasm when observed in whole mounts with light microscopy [20, 21], we suggest that the parasites may actually be on the surface of the cell in many cases, and that this may account for the apparent increased number of amastigote-like cells counted inside Vero cells in previous studies [20, 21]. Observing SEM images of PtK2 and Vero cells after 48 h, it is clear that many parasites became embedded in the cell membrane. This interaction was observed with higher frequency in Vero cells and could account for the large number of apparent intracellular parasites counted [21]. This type of interaction was noticed in *T. cruzi* amastigotes generated *in vitro* interacting with HeLa and Vero cells where SEM micrographs show microvilli-like structures that engulf the amastigotes upon attachment after blocking dynamin in co-cultures with peritoneal macrophages [28]. HeLa cells produced cup-like extensions to engulf amastigotes and Vero cells produced actin-rich crater-like indents where amastigotes were attached [44]. The SEM images in Procopio et al. [44] and Barrias et al. [28] show cup-shaped structures creeping up the sides of the parasite and crater-like areas where the parasites had become embedded in the cell membrane, changes that were also seen to some extent in the current study with *T. copemani*. However, most amastigote-like parasites of *T. copemani* were not engulfed and SEM images revealed they were in the process of degrading. External amastigote-like parasites (even dying ones with damaged membranes) appear to be within the cells in brightfield images due to the permeabilising effect of methanol, which collapses the cells making them appear two-dimensional. The significance of the partial engulfing of amastigote-like cells explains why the parasites were not washed away during processing despite not being inside the cell cytoplasm, and why there appears to be so many intracellular parasites in Vero cells at 48 h. The number of intracellular parasites then decreased after 48/72 h [21], which could be explained by the continued division of healthy cells while the amastigote-like parasites became completely degraded. The cells were washed in fresh liquid media supplemented with 10% FCS after parasite incubation and recovered quite fast, as demonstrated in live-cell time-lapse data from the current study. Amastigote transformation *in vitro* can be triggered by a reduction in pH and does not require trypomastigote entry into the cell cytoplasm [45], which could explain the appearance of the amastigote like cells in *T. copemani*. However, further investigation is required to conclusively demonstrate under what circumstances *T. copemani* can be internalised into the cell cytoplasm, as observed in TEM micrographs.

*Trypanosoma cruzi* cell infection methodologies show great variability, which makes it difficult to compare infection rates as the type of host cell and strain of *T. cruzi* are variable factors in the intracellular life-cycle [25, 46]. However, taking this variability into consideration, *T. cruzi* 10R26 strain (TcIIa) infects marsupial cells in a similar manner to other mammalian cells *in vitro* with many cells containing dividing parasites and releasing new metacyclic trypomastigotes after four days. Other studies often use higher numbers of parasites such as 5 × 10^7^ parasites per well [40], which is 25 times more parasites than used in this study, accounting for the higher infection rates observed in other *T. cruzi* experiments [46]. The lower number of parasites used for infection in this study allowed the observation of cells over a longer time period with marsupial cells. The only time Australian marsupials were exposed to *T. cruzi* under laboratory settings, a high mortality rate was observed in infected possums [10]. Although a natural *T. cruzi* infection acquired by marsupials in the wild may be initially devastating due to the lack of immunity to cope with infections, the animals could still transmit and amplify the pathogen as it appears to invade and replicate normally in their cells. However, it is not possible to predict exactly what would occur under natural *in vivo* conditions from *in vitro* experiments. It can be speculated that if *T. cruzi* was to become established in Australia it would be detrimental to both human and wildlife populations.

Without the presence of an immune response *in vitro*, stressed cells provide seemingly excellent conditions for cell entry. If *T. copemani* cannot enter and replicate under these conditions as does *T. cruzi*, it is unlikely they could do so in a healthy animal. This is consistent with research on other intracellular trypanosomes, apart from *T. cruzi*, including *T. theileri* [16], *T. erneyi* [17] and *T. rangeli* [17–19], which have also been observed invading cells *in vitro* but not *in vivo*. The negative reaction from cells observed in this study is not expected from intracellular pathogens interacting with host cells. Intracellular pathogens like *T. cruzi*, as well as *Leishmania*, *Cryptosporidium*, *Theileria*, *Toxoplasma* and *Plasmodium*, all have the ability to inhibit parts of the apoptotic pathway [47]. By controlling mammalian cell processes, parasites like *T. cruzi* can facilitate infection and replication in healthy cells [48]. Although there is no indication *T. copemani* invades cells like *T. cruzi*, the detrimental interaction observed between *T. copemani* and mammalian cells indicates that further investigation needs to be performed, due to the likelihood of an impact on the host. We cannot rule out the possibility *T. copemani* enters cells in an immunosuppressed host. Many native marsupials in Australia are vulnerable or endangered and under stress. While *T. copemani* could contribute to ill health in the woylie [20, 21, 49–51], whether it causes clinical trypanosomiasis remains unknown. Previous ecological evidence suggests that *T. copemani* may have been involved in, but not directly responsible for, the decline of the woylie [20, 23, 43, 52]. The remaining woylie populations could be stressed and thus immunosuppressed [49], which may affect the resident parasite population and normal host-parasite relationships. This perhaps allows cell invasion by a parasite that is a facultative intracellular pathogen. As *T. copemani* does damage the cells *in vitro*, this study suggests that it would be possible for these parasites to attach to cells in the host. It cannot be ruled out that *T. copemani* and its interaction with marsupials is detrimental to the host due to attachment to red blood cells, causing anaemia, which has been proposed before [50].

Despite the implications of trypanosome inflicted disease in Australian wildlife in the past, there is no discerning histology to date [8, 20, 50]. Amastigote-like structures were observed inside woylie heart sections from an animal infected with *T. copemani* G2 [20]. However, due to the extreme difficulty in obtaining adequately preserved tissue samples from these vulnerable and endangered host species it has not yet been possible to investigate this further. Wild animal carcases recovered by conservation agencies are often either frozen or have been dead for too long before discovery to be of use for microscopy examinations at the resolution required to identify trypanosomes. Additionally, while PCR analysis can indicate the presence of parasites in the animal, it does not allow the presence of intracellular parasites to be determined. To date, no research has been conducted on *T. copemani* antigens, or protein expression. Furthermore, the processes and molecules involved in mammalian cell invasion in *T. cruzi* are so varied it is not feasible to compare mechanisms/molecules involved in *T. copemani* invasion at present. Morphological responses have been investigated in this study to investigate the parasite life-cycle and, based on previous observations, whether it involves an intracellular stage. Using genetic inhibition could be utilised to explore the similarities between *T. cruzi* and *T. copemani*. For example, the effects that host-cell inhibition of apoptosis, actin remodelling and endocytosis have on infection dynamics are well established in *T. cruzi* and this could be explored in *T. copemani* [28, 53–55].

The cell-parasite interaction observed raises many issues surrounding trypanosome intracellular behaviour. It should be considered whether intracellular behaviour in *T. copemani* is an evolutionary remnant of the past and is a completely different strategy for trypanosomes that are facultatively intracellular, or whether it is a transitory defence strategy by *T. copemani*. Australian trypanosomes may once have possessed the ability to enter cells and divide, but if this has been lost they no longer need to escape the host immune system in order to replicate.

## Conclusions

*Trypanosma copemani* is not an obligate intracellular parasite and does not show similarities to *T. cruzi in vitro*. However, *T. copemani* does adversely affect cell health and there are behavioural differences between genotypes (G1 and G2) *in vitro*. The results of the present study indicate that a more traditional trypanosome life-cycle that involves two morphological forms, including epimastigotes in the invertebrate host and trypomastigotes in the vertebrate host, is more likely for *T. copemani*. Future studies in this area should focus on acquiring histology from the marsupial hosts, investigating the possibility that Australian trypanosomes cause anaemia, and investigating potential trypanosome vectors in Australia. Future investigation should also focus on increasing the understanding of the *T. copemani* life history and the differences between the different genotypes (G1 and G2).

## Additional files

Additional file 1: **Figure S1. a** *Trypanosoma copemani* infecting potoroo kidney epithelial (PtK2) cells *in vitro* fixed after 72 h. Parasites and cells are stained with propidium iodide (PI). Cell nuclei and parasite nuclei are easily differentiated due to the difference in their size. The left arrow points to the small nuclei of the parasite and the right arrow points to the larger nuclei of the cell. Channels are split into DIC, PI, and both channels merged. **b-d** *Trypanosoma copemani* infecting potoroo kidney epithelial (PtK2) cells *in vitro* live-cell time-lapse 24 h after infection. Parasites and cells are stained with PI showing easily distinguishable nuclei (arrows). Images show merged channels. Objective used in all images was 23×. *Scale-bar*: 20 μm. (TIF 16781 kb)

Additional file 2: **Figure S2.** *Trypanosoma copemani* G1 incubated with potoroo epithelial kidney (PtK2) cells. **a** G1 exhibiting internal amastigotes inside PtK2 cell. **b** G1 amastigote inside PtK2 cell. All images are stained with Diff-Quik. *Scale-bars*: 20 μm. (TIF 955 kb)

Additional file 5: **Figure S3.** Time-lapse live-cell video demonstration of the recovery of potoroo kidney epithelial cells after being washed once with 1× PBS and resuspended in full MEM media following incubation with *Trypanosoma copemani* G2 trypomastigotes. **a** After 24 h, **b** 36 h, **c** 48 h. *Scale-bar*: 50 μm. (TIF 872 kb)

## Abbreviations

SEM: Scanning electron microscopy
TEM: Transmission electron microscopy
G1: *Trypanosoma copemani* genotype 1
G2: *Trypanosoma copemani* genotype 2
PtK2: Potoroo kidney epithelial cells
PI: Propidium iodide
