# Supplementary figures and images for: The marsupial trypanosome Trypanosoma copemani is not an obligate intracellular parasite, although it adversely affects cell health
Source: Parasit Vectors. 2018 Sep 20;11:521. doi: 10.1186/s13071-018-3092-1 (PMC6148770; doi:10.1186/s13071-018-3092-1)

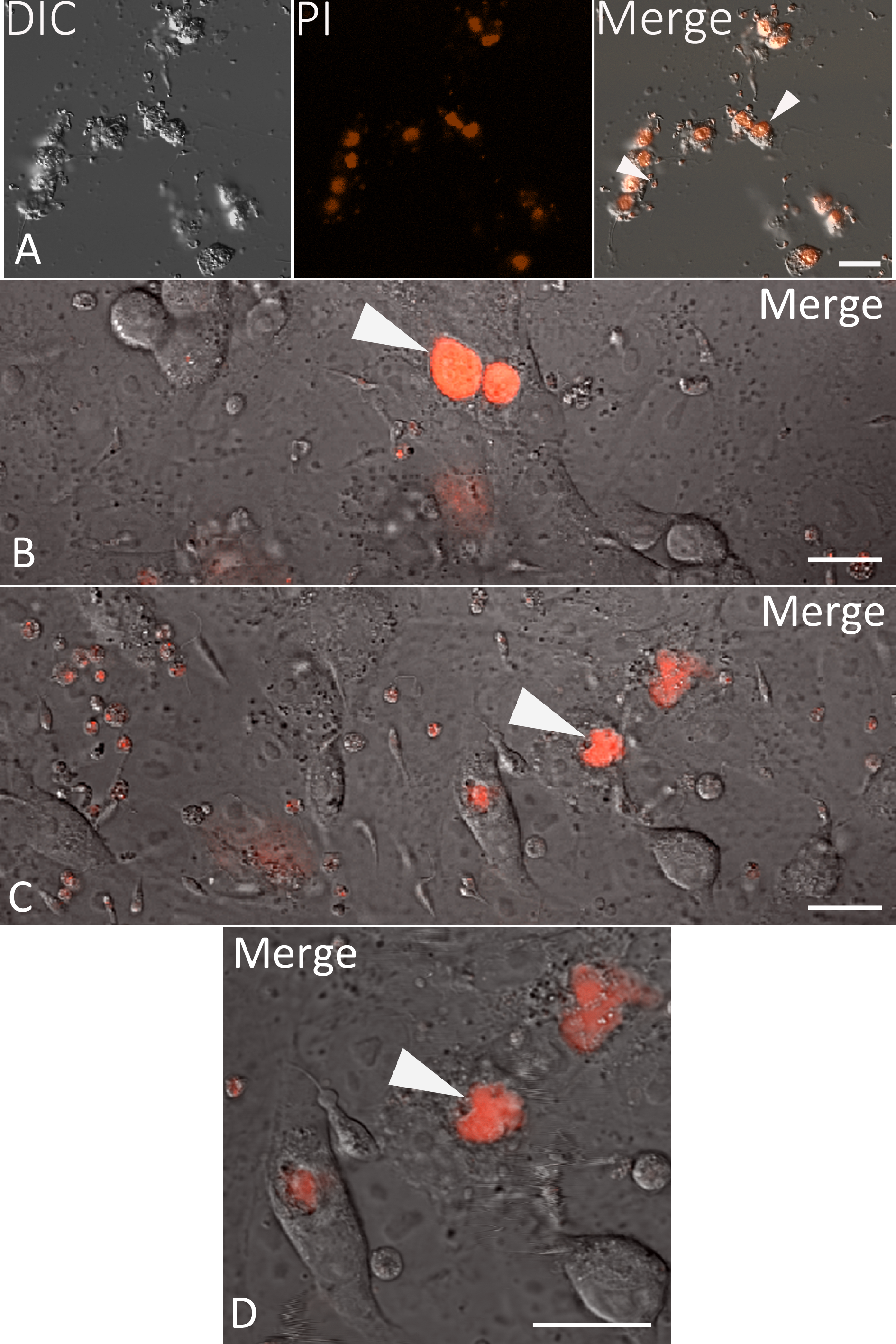

Supplement: Supplementary file 4 — Figure S1. a Trypanosoma copemani infecting potoroo kidney epithelial (PtK2) cells in vitro fixed after 72 h. Parasites and cells are stained with propidium iodide (PI). Cell nuclei and parasite nuclei are easily differentiated due to the difference in their size. The left arrow points to the small nuclei of the parasite and the right arrow points to the larger nuclei of the cell. Channels are split into DIC, PI, and both channels merged. b-d Trypanosoma copemani infecting potoroo kidney epithelial (PtK2) cells in vitro live-cell time-lapse 24 h after infection. Parasites and cells are stained with PI showing easily distinguishable nuclei (arrows). Images show merged channels. Objective used in all images was 23×. Scale-bar: 20 μm. (TIF 16781 kb) [file 13071_2018_3092_MOESM1_ESM.tif]

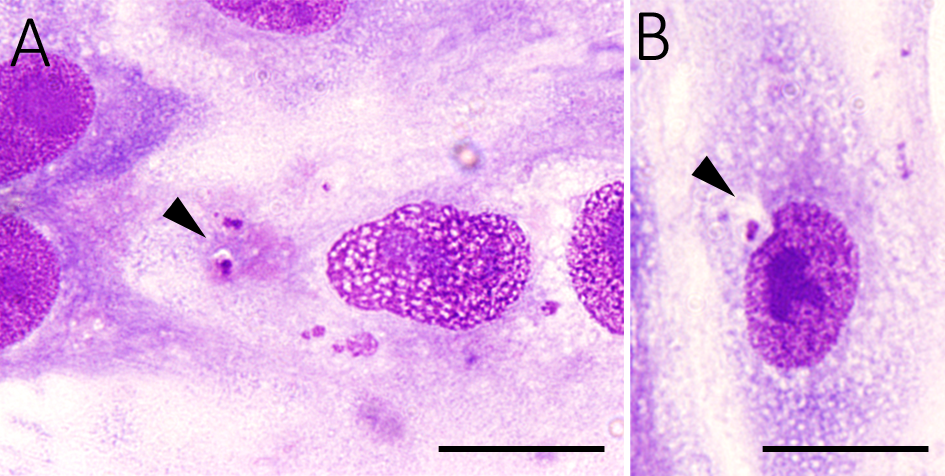

Supplement: Supplementary file 5 — Figure S2. Trypanosoma copemani G1 incubated with potoroo epithelial kidney (PtK2) cells. a G1 exhibiting internal amastigotes inside PtK2 cell. b G1 amastigote inside PtK2 cell. All images are stained with Diff-Quik. Scale-bars: 20 μm. (TIF 955 kb) [file 13071_2018_3092_MOESM2_ESM.tif]

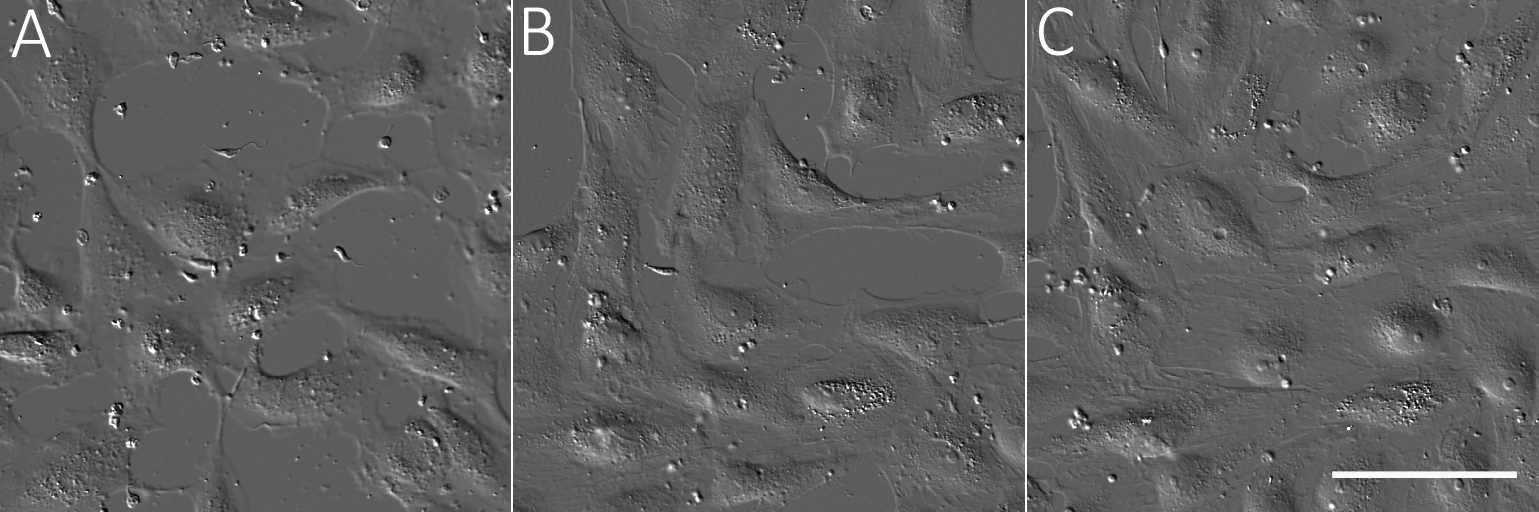

Supplement: Supplementary file 6 — Figure S3. Time-lapse live-cell video demonstration of the recovery of potoroo kidney epithelial cells after being washed once with 1× PBS and resuspended in full MEM media following incubation with Trypanosoma copemani G2 trypomastigotes. a After 24 h, b 36 h, c 48 h. Scale-bar: 50 μm. (TIF 872 kb) [file 13071_2018_3092_MOESM5_ESM.tif]
